# Retraction: Economic impact of a vision-based patient monitoring system across five NHS mental health trusts

**DOI:** 10.1371/journal.pdig.0001592

**Published:** 2026-07-15

**Authors:** The PLOS Digital Health Editors

Following publication of this article [1], concerns were raised about the selection of parameter values used in the economic model evaluating the impact of the vision-based patient monitoring system (VBPMS), Oxevision, developed and marketed by Oxehealth. Model input data for clinical parameters were gathered from a mix of five published and unpublished before-and-after studies performed at different NHS sites; analysis of these study data was carried out by Oxehealth. In post-publication discussions of this matter, the authors stated that they had not been given access to the full clinical study data.

PLOS reassessed the article and additional information provided by the authors with support of a member of the *PLOS Digital Health* Editorial Board and another expert. This editorial review concluded that:

- Aspects of the study design were not adequately justified, including
  - Inconsistencies in data sources for clinical parameter model inputs,
  - Selection of some specific assumptions and inputs,
  - Data restrictions such that the authors did not have access to the clinical data from all five studies that informed the model inputs;
- There may have been bias in the selection of model input data, and the risk of bias in model input data in view of competing interests was not adequately addressed by processes used to evaluate data sources or in the analytical approaches to evaluate uncertainty;
- It is not possible to independently evaluate the validity of the model input selections without the complete underlying data which were not made available to the authors, editors, or reviewers;
- The article [1] did not report a sensitivity analysis or details of the Excel model developed in the study; these items were provided for post-publication editorial review and a consulted expert identified additional concerns about inadequate support for the base case estimate and the ranges for each parameter in the provided sensitivity analyses.

The above issues call into question the reliability of the reported results and the article’s adherence to PLOS policies. Therefore, the *PLOS Digital Health* Editors retract this article [1].

Note: In following up on this matter, it was noted that in Table 4 of S1 Appendix in the Supporting Information files of [1] there is an error in reference 6. The correct reference, cited in [1] as Reference 7 of the main article References section, is: Ndebele F, Wright K, Gandhi V, Bayley D (2024) Non-contact monitoring to support care in acute inpatient mental health. Journal of Mental Health 33(3): 320–325. https://doi.org/10.1080/09638237.2023.2245882

Additionally, the Competing Interests statement for [1] did not state that Oxehealth developed and markets the Oxevision system, which is the subject of the evaluation, although this link between Oxevision and Oxehealth is made clear in the article’s Introduction.

All authors agreed with the retraction.
